# Study of Rectal Diazepam in Prevention of Simple Febrile Convulsions Recurrence

**Published:** 2011-06-01

**Authors:** M M Taghdiri, A Heidari, M Mojarrad, M Fallah

**Affiliations:** 1 Department of Pediatrics, Besat Hospital, Hamedan University of Medical Sciences, Hamedan, Iran

**Keywords:** Febrile convulsions, Rectal dazepam, Recurrence

Dear Editor,

Febrile convulsions, the most common seizure disorder during childhood are age dependent and are rare before 9 months and after 5 years of age.[1][2] Approximately 30-50% of children have recurrent seizures with later episodes of fever and a small minority has numerous recurrent febrile seizures.[3][4] The routine management of a normal infant with simple febrile convulsions includes a careful search for the cause of the fever and reassurance and education of the parents.[5][6]

In this randomized clinical trial, 80 children aged 9 months to 5 years with simple FC referred to Pediatric Neurology Clinic in 2003 were divided into two 40 patients' random groups. We advised one group using rectal diazepam (0.5 mg/kg) in the form of gelnamed Diastat in addition to wet sponge and acetaminophen while they were febrile and another group only wet sponge and acetaminophen. They were followed for a one year period regarding the seizures recurrence. Data were collected by special charts including age, gender, seizure type, the reason of fever and family history. Statistical analysis of data was performed using SPSS software by Chi Square and t-tests. A p value <0.05 was considered significant.

The rate of recurrence in first group that did not receive any rectal diazepam was 37.5% (15 cases) and in second group was 27.5% (11cases). Of 40 patients in first group, 20 cases (50%) were male and 20cases (50%) were female. In another group who received rectal diazepam, 28 patients (70%) were male and 14 cases were female (30%). The mean age in the first group was 27.2 months and in second group was 29.2 months. Most of patients were in the range of 9-18 months old age group (43.8%).

In the first group, 9 cases (22.5%) had family history of simple febrile convulsions and in the other group 6 cases (15%) had (p>0.05). The rate of recurrence without consideration of positive family history (for elimination of intervening variable) in the first group was 11 cases (35.5%) and in the other group was 9 cases (26.5%) that fortunately was ignorable in our final conclusion (p>0.05).

The duration of seizures in 70% of patients in the first group and 80% of cases in second group was approximately 5 minutes, In 11% of the first and 12.5% of the second group was 10 minutes and in 2.5% of the first and 7.5% of the second group was 15 minutes (p>0.05). Most of recurrences occurred in the range of 19-36 months old age group (37.1%), and other ranges were 31% in 9-18 months, and 25% in 3-5 years old age groups (p>0.05). The recurrence of seizure during the first 3 months after primary seizure was 34.6%, 26.9% during the second 3 months, 23% during the 3rd three months and 15.3% in the 4th 3months after the primary seizures (p>0.05)(Table 1).

Over the last few years, there has been a fair degree of consensus as to the management of a child who has experienced his or her first febrile seizure.[7] Study of Camfiled showed that the rate of FC recurrence to be less when using rectal diazepam versus a placebo.[8] Rectal diazepam is well tolerated for acute repetitive seizures and can be administered at home by trained caregivers as a preferable way.[9][10]

In conclusion, our findings showed that prescription of rectal diazepam at the time of fever may reduce the risk of recurrence of seizures in simple febrile convulsions.

**Table 1:** Comporison of two groups regarding the gender, rate of recurrence, mean age, family history and duration of seizure.

	**Treatment group (n=40) **	**Control group (n=40) **	**P value **
Gender	Male=28	Male=20	0.06
	Female=14	Female=20	
Rate of recurrence	11	15	0.43
Mean age	29.2	27.2	0.56
Family history of FC	6	9	0.39
Duration of seizure	5 min=32	5 min=28	0.27
	10 min=5	10 min=11	
	15 min=3	15 min=1	
